# Knowledge, beliefs and practices towards HIV/AIDS among adolescents in India: A scoping review protocol

**DOI:** 10.1371/journal.pone.0280985

**Published:** 2023-02-14

**Authors:** Pranita Patsani, Jayashree Parida, Arpita Panda, Susangita Jena, Swati Sukalyani Behera, Abinash Pradhan, Prasanna Kumar Patra, Sanghamitra Pati, Harpreet Kaur, Subhendu Kumar Acharya

**Affiliations:** 1 ICMR- Regional Medical Research Centre, Bhubaneswar, Odisha, India; 2 Department of Anthropology, Utkal University, Bhubaneswar, Odisha, India; 3 Division of ICMR, Division of Epidemiology and Communicable Diseases (ECD-Tribal Health), New Delhi, India; Pokhara University, NEPAL

## Abstract

**Introduction:**

Acquired Immune Deficiency Syndrome (AIDS) is a major health concern among Indian adolescents (10–19 years). Indian adolescents lack adequate knowledge about HIV/AIDS and adopt wrong practices. The present scoping review aims to understand the status of knowledge, beliefs and practices among Indian adolescents about HIV or HIV/AIDS. The present study will also focus on their understanding and the source of knowledge and awareness regarding HIV/AIDS.

**Methodology:**

The recommendations of Arksey and O’Malley in 2005, Levac et al. in 2010 and The Joanna Briggs Institute Reviewers’ manual in 2015 to conduct a systematic scoping review will be employed. The review questions, eligibility criteria and search strategy for this study will be ensured by the Population, Concept and Context (PCC) strategy. The Preferred Reporting Items for Systemic Reviews and Meta-Analyses extension for Scoping Reviews (PRISMA-ScR) will be used for reporting of this scoping review. The methodological quality of all the relevant studies will be assessed by the Mixed Methods Appraisal Tool (MMAT), Version- 2018. Literature search will be carried out by using electronic databases such as PubMed, APA PsycInfo, EMBASE, and Google Scholar. Cross-references will be used to extract additional studies.

**Ethics and dissemination:**

As the planned study is based on secondary data and doesn’t involve human and animal subjects, there is no requirement for formal ethical approval.

**Strengths and limitations of the study:**

- The planned scoping review is the first of such study on the topic of HIV/AIDS among adolescent population in India as per the electronically available literature.
- There will be quality assessment of all the included studies.
- A thorough search strategy will be conducted as per the recommendations of the experts in scoping review search.
- On limitation side, the planned study will only utilize electronically available resources/evidences that are written in English language.
- The planned evidence based study will be of much help for the healthcare professionals and policy makers.

## Introduction

Adolescent folk is 1.2 billion worldwide which constitute 18 percent of the total world population [1]. With 253 million adolescent individuals, India is the most populated country in terms of adolescents in the world as there is one adolescent in every five person in the country [2].

Adolescence is generally full of inquisitiveness and hence adolescents often try to explore new things. During this phase in life, they also attain puberty and reach sexual maturity which leads to increased fascination with various sexual activities and hence contributing to the risk for HIV transmission. Human Immunodeficiency Virus (HIV) that causes Acquired Immunodeficiency Syndrome (AIDS) poses a great threat to healthcare and a public health issue globally [3]. Unprotected sexual activity is a predominant mode of transmission of HIV/AIDS [4]. During this phase, adolescents also get habituated with other health risk behaviors like drug abuse (one of which is intravenous drug injection) which is another reason of HIV/AIDS transmission. A report by WHO (2021) says that HIV infection is the sixth major cause of adolescent mortality all over the world [5].

According to a recent report published by United Nations Children’s Fund (UNICEF) (2020), an estimated 1.75 million adolescents are living with HIV by the end of 2020 [6]. Also, in 2020 itself, 150,000 adolescents newly acquired HIV and 32,000 adolescents died due to HIV throughout the world [6]. Given the fact that immediate preventive measures are not taken for HIV/AIDS prevention, there will be approximately 80 adolescent deaths per day due to HIV/AIDS globally by the year 2030 [7]. India reports the highest number of adolescent HIV infection cases in South Asia [8]. In the context of the increasing burden of HIV/AIDS among the adolescents, several studies around HIV/AIDS have been undertaken in different parts of the country to generate evidence and necessary data around HIV associated various aspects among Indian population including adolescents. A report published by UNICEF (2021) says that in the year 2016, the presence of comprehensive and correct knowledge about HIV among male and female adolescents aged 15–19 years in India is highly limited with 28.20% and 18.50% respectively [6]. Since knowledge, beliefs and practices regarding HIV/AIDS are important parameters in the fight against the disease [9], adolescents must be endowed and empowered with knowledge regarding safe practices and high risk behaviors.

In India, most studies among adolescents have been undertaken to understand their knowledge, attitude and practices (KAP) regarding HIV/AIDS [10,11]. However, there is a vacuum in comprehensive reviews around such aspects among adolescents around HIV/AIDS associated knowledge in the country. With our preliminary analyses, we observed a significant gap in evidence synthesis and systematic review around HIV/AIDS among adolescents throughout India. To our best knowledge, the planned scoping review is going to be the first of its kind in Indian context to assess the status of adolescents’ knowledge, awareness, beliefs and practices by taking a large number of studies into consideration. This scoping review will do it among adolescents uninfected with HIV, by exploring their sources of information and individual attitude towards persons living with HIV/AIDS. It will also study the psychosocial perceptions of adolescents living with HIV like, for example, in a study conducted by Gupta et al. [12] it was found that 9 per cent of adolescents are afraid that they may be removed from their school/house/society while 11 per cent feel worthless and inferior because they are infected with HIV. India is going to be benefitted socially, economically and politically as well if these adolescents are safe, healthy, educated and equipped with knowledge but they are going to face repercussions if it happens otherwise [3].

In the above context, it is important to mention that in spite of several studies reporting about the status of knowledge, beliefs and practices of adolescents regarding HIV/AIDS in many of the Indian states, the national level estimation is inadequate. In India, there is no recent scoping or systematic review available, particularly focusing on the status of awareness regarding HIV/AIDS among adolescents. The scoping review is here being conducted to identify the knowledge gaps, clarify the concepts and map the available evidence [13]. Therefore, the present scoping review aims to investigate the status of knowledge, prevailing attitudes, beliefs and practices regarding HIV/AIDS among the adolescent population in India and also to discuss the source of knowledge regarding the same.

## Objectives

1. To determine the status and source of knowledge of HIV/AIDS among Indian adolescents.
2. To find out the beliefs and attitudes of Indian adolescents towards people living with HIV/AIDS.

## Methodology

Firstly, online database and protocol registration websites like Figshare and Open Science Framework were checked for the availability of any scoping review around the defined objectives. In the context of the unavailability of any previously published scoping review on knowledge, beliefs and practices of adolescents towards HIV/AIDS in India, the planned study was designed.

The Arksey and O’Malley’s (2005) [14], Levac et al. (2010) [15] and The Joanna Briggs Institute Reviewers’ manual (2015) [16] recommendations will be used to conduct this systematic scoping review focused on adolescents’ knowledge, beliefs and practices regarding HIV/AIDS.

The Preferred Reporting Items for Systematic reviews and Meta-Analyses extension for Scoping Reviews (PRISMA-ScR) checklist [17] will be followed to report the scoping review while we have used PRISMA-P to prepare the checklist in the present protocol as per the recommendation [18]. The PRISMA-P checklist is given in **S1 File**. The research questions, search strategy and eligibility criteria will be conducted based on the Population, Concept and Context (PCC) strategy. The Mixed Methods Appraisal tool (MMAT), Version- 2018 will be used to appraise the quality of empirical studies including qualitative, quantitative and mixed method studies [19]. The tool is designed to include qualitative, quantitative randomized trials, quantitative non-randomized, quantitative descriptive and mixed method studies [20]. There are different quality assessment criteria for each study design. MMAT is only designed for the purpose of quality assessment of studies and not to exclude studies based on the quality assessment score. Thus, it will be helpful in presenting a comprehensive overview of the available evidence. The quality assessment score will be used to understand the overall quality of the studies undertaken and data published [19].

The Arksey and O’Malley framework that will be included in the planned study are as follows:

1. Identifying the research questions
2. Retrieving relevant studies
3. Selection of studies
4. Charting data
5. Collating, summarizing, and reporting evidence

### Identifying the research questions

The main research question will be: What is the status of knowledge and awareness regarding HIV/AIDS among the adolescents of India?

The Sub review questions will be as follows:

- What are the possible sources of knowledge regarding HIV/AIDS for Indian adolescents?
- What are the beliefs and attitudes towards HIV/AIDS patients among Indian adolescents?

### Retrieving relevant studies

An exhaustive search will be conducted for potentially eligible articles in the following databases: EMBASE (https://www.embase.com/search/quick), PubMed (https://pubmed.ncbi.nlm.nih.gov) and APA PsycInfo (https://www.apa.org/pubs/databases/psycinfo). Grey literature search will be performed through ResearchGate (https://www.researchgate.net), Shodhganga (https://shodhganga.inflibnet.ac.in) and Google Scholar (https://scholar.google.com; India) to obtain relevant references. In addition to these, other governmental and non-governmental websites such as World Health Organization, UNICEF, National Family and Health Survey, National Institute of Health and Family Welfare, National AIDS Control Organization, Abstracts archive (https://www.abstract-archive.org) etc. will also be searched for other grey literature in the area. Search strategies will be created with the help of people having expertise in scoping review searching. Relevant keywords will be finalized according to the Population, Concept and Context (PCC) strategy before searching. The database search will be done using the following keywords: “HIV AIDS”, “HIV infection”, “Acquired immunodeficiency syndrome”, “STDs adolescents”, “India”, “Adolescent”, “Teenager”, “Knowledge”, “Health Attitude”, “Health Practices”, “Health Beliefs”, “New Delhi”, “Maharashtra”, etc. Different search terms will be used in each databases for the need of including both free and controlled vocabulary (i.e. Medical Subject Heading terms in PubMed, Thesaurus terms in APA PsycInfo and Keywords in EMBASE). Boolean terms (AND/ OR) will be used to separate the keywords in specific databases. The searching strategy to be used for PubMed and EMBASE electronic databases is given in **S2 File**. Search results will be limited to articles published in recent decades between the years 2000–2022 as the preliminary searches revealed limited publications before 2000. Reference lists of included articles and relevant review articles will be screened for any additional references that may be eligible for inclusion in the planned study.

### Selection of studies

The inclusion and exclusion criteria will be created based on the PCC strategy.

#### Inclusion criteria

- **Population**: The adolescent group aged from 10–19 years.
- **Concept**: Knowledge, beliefs and practices regarding HIV/AIDS among the adolescents.
  We define knowledge regarding HIV/AIDS as the awareness and information of HIV/AIDS related facts among adolescents for example, with respect to its symptoms, modes of transmission and prevention.
  Similarly, by beliefs regarding HIV/AIDS we mean the underlying socio-cultural perceptions of an adolescent towards HIV/AIDS and HIV-positive individuals; and practices are defined as the discriminatory and sympathizing behaviors and attitudes of adolescents towards HIV-positive individuals.
- **Context**: India
- The studies done in and between the year 2000–2022 will be included in the planned review.
- All the studies that are published in English language only will be selected as majority of research publications are in English.
- Studies with empirical data on status of knowledge, beliefs and practices such as cross-sectional studies, interventional studies, case-controlled studies.

#### Exclusion criteria

- Studies undertaken prior to the year 2000 or after the year 2022.
- Studies done in countries other than India.
- Studies from which data extraction for the desired age range cannot be conducted.
- Papers on Sexually Transmitted Diseases (STDs) from which data extraction for HIV/AIDS cannot be undertaken.
- Papers with no appropriate original data will be excluded, to avoid duplication of data.

Guided by the already defined inclusion and exclusion criteria, database search and title screening will be conducted. The reviewers will screen the abstracts and full text articles in parallel. In this context, if any disagreement among the reviewers arises, it will be resolved by discussing the disagreement to arrive at a consensus or by referring to the third reviewer, if no consensus is reached. All the stages for selection of the relevant studies will be presented in the flow diagram as prescribed in Preferred Reporting Items for Systemic Review and Meta-Analysis extension form Scoping Reviews (PRISMA-ScR).

### Charting the data

A thorough reading of the included studies will be done for the extraction of relevant data. A predefined data extraction summary table will be developed using Ms-Excel spreadsheet which presents all the key information of the selected studies. This spreadsheet will include the following information:

- Study title
- Author’s information
- Year of the study
- The published journal
- Region(s)
- Age
- Sexes
- Sample size
- Sampling methods
- Status of knowledge assessed through knowledge about the modes of transmission and prevention of HIV/AIDS among the adolescents.
- Status of beliefs and misbeliefs regarding HIV/AIDS and people living with HIV/AIDS among the adolescents.
- Status of practices (both stigmatizing and sympathizing attitudes) towards people living with HIV/AIDS among the adolescents

Before finalization, feedbacks from the investigators will be considered to update the data extractions from the included studies.

### Collating, summarizing, and results

As the aim of the proposed scoping review will be to collect the existing evidence on the status of knowledge, beliefs and practices regarding HIV/AIDS among the adolescents, summarize the results as reported in the included studies and identify gaps for further research. All the processed evidence as discussed above will proceed to summarization, analysis, and the final report will be prepared with a focus on the study’s outcome of interest (Status of knowledge, beliefs and practices regarding HIV/AIDS among adolescents of India). The findings will be analyzed through thematic content analysis and will take a narrative approach. Other emerging themes will also be reported.

#### Patient and public involvement

No patients will be involved.

#### Ethics and dissemination

As the planned study is based on secondary data and doesn’t involve human and animal subjects, there is no requirement for formal ethical approval.

## Discussion

Adolescence is a distinct age group (10–19 years) with complex needs because of the physical and psychological development during puberty and the transitional phase in life before acquiring adulthood [21]. At this stage, they are at their hormonal peak, with the want to look their best, and perform the best in order to establish their identity in society due to which they don’t refrain themselves from performing high risk behaviors [21]. The risk of exposure to HIV/AIDS is the highest among the adolescent group which is a major health challenge for the country. The risk of getting infected with HIV is strongly related with peer pressure and drug abuse behavior which means that adolescents under the influence of peers are more likely to take drugs, have multiple sexual partners and perform unsafe sex [22]. Therefore, creating awareness among the adolescents and their peer groups can help in preventing HIV/AIDS among them. Also, the status of awareness of HIV among adolescents is quite alarming [11,23,24]. Hence there is an urgent need to assess the status of correct knowledge among adolescents which can help to create the required awareness to fight against the HIV epidemic well.

This proposed scoping review will provide an unprecedented overview of the available literature on HIV among Indian adolescents. The proposed scoping review will systematically map the evidence to identify the primary research issues on HIV/AIDS in India. The potential gaps in this issue will provide crucial inputs to healthcare agencies and policymakers to propose new research questions so as to improve adolescent health in India. The present review will be an important reference for other low and middle-income countries where HIV/AIDS is a major concern among adolescents. According to UNAIDS estimates, 71% of the estimated 2.1 million adolescents living with HIV lived in 10 countries that included nine sub-Saharan African countries and India [25].

## Supporting information

S1 File PRISMA-P checklist. (DOCX)

S2 File Search strategy for PubMed and EMBASE electronic database. (DOCX)
