# Supplementary material for: Knowledge, beliefs and practices towards HIV/AIDS among adolescents in India: A scoping review protocol
Source: PLoS One. 2023 Feb 14;18(2):e0280985. doi: 10.1371/journal.pone.0280985 (PMC9928123; doi:10.1371/journal.pone.0280985)
Supplement: S2 File — (DOCX) [file pone.0280985.s002.docx]

**Supplementary File**

**1: Draft of search strategy to be used in PubMed electronic database**

| **Components** | **Search items** | **Results** |
| --- | --- | --- |
| #1 | **knowledge:** "knowledge"[MeSH Terms] OR "knowledge"[All Fields] OR "knowledge s"[All Fields] OR "knowledgeability"[All Fields] OR "knowledgeable"[All Fields] OR "knowledgeably"[All Fields] OR "knowledges"[All Fields] | 965323 |
| #2 | **health beliefs:** ("health"[MeSH Terms] OR "health"[All Fields] OR "health s"[All Fields] OR "healthful"[All Fields] OR "healthfulness"[All Fields] OR "healths"[All Fields]) AND ("belief s"[All Fields] OR "culture"[MeSH Terms] OR "culture"[All Fields] OR "belief"[All Fields] OR "beliefs"[All Fields]) | 259044 |
| #3 | **health attitude:** "attitude to health"[MeSH Terms] OR ("attitude"[All Fields] AND "health"[All Fields]) OR "attitude to health"[All Fields] OR ("health"[All Fields] AND "attitude"[All Fields]) OR "health attitude"[All Fields] | 604894 |
| #4 | **health practices:** ("health"[MeSH Terms] OR "health"[All Fields] OR "health s"[All Fields] OR "healthful"[All Fields] OR "healthfulness"[All Fields] OR "healths"[All Fields]) AND ("practicability"[All Fields] OR "practicable"[All Fields] OR "practical"[All Fields] OR "practicalities"[All Fields] OR "practicality"[All Fields] OR "practically"[All Fields] OR "practicals"[All Fields] OR "practice"[All Fields] OR "practice s"[All Fields] OR "practiced"[All Fields] OR "practices"[All Fields] OR "practicing"[All Fields]) | 837602 |
| #5 | **knowledge attitude:** ("knowledge"[MeSH Terms] OR "knowledge"[All Fields] OR "knowledge s"[All Fields] OR "knowledgeability"[All Fields] OR "knowledgeable"[All Fields] OR "knowledgeably"[All Fields] OR "knowledges"[All Fields]) AND ("attitude"[MeSH Terms] OR "attitude"[All Fields] OR "attitudes"[All Fields] OR "attitude s"[All Fields]) | 181235 |
| #6 | **knowledge attitude practice:** ("knowledge"[MeSH Terms] OR "knowledge"[All Fields] OR "knowledge s"[All Fields] OR "knowledgeability"[All Fields] OR "knowledgeable"[All Fields] OR "knowledgeably"[All Fields] OR "knowledges"[All Fields]) AND ("attitude"[MeSH Terms] OR "attitude"[All Fields] OR "attitudes"[All Fields] OR "attitude s"[All Fields]) AND ("practicability"[All Fields] OR "practicable"[All Fields] OR "practical"[All Fields] OR "practicalities"[All Fields] OR "practicality"[All Fields] OR "practically"[All Fields] OR "practicals"[All Fields] OR "practice"[All Fields] OR "practice s"[All Fields] OR "practiced"[All Fields] OR "practices"[All Fields] OR "practicing"[All Fields]) | 147598 |
| #7 | **health knowledge attitudes practice:** "health knowledge, attitudes, practice"[MeSH Terms] OR ("health"[All Fields] AND "knowledge"[All Fields] AND "attitudes"[All Fields] AND "practice"[All Fields]) OR "practice attitudes health knowledge"[All Fields] OR ("health"[All Fields] AND "knowledge"[All Fields] AND "attitudes"[All Fields] AND "practice"[All Fields]) OR "health knowledge attitudes practice"[All Fields] | 131022 |
| #8 | **hiv aids:** "curr opin hiv aids"[Journal] OR ("hiv"[All Fields] AND "aids"[All Fields]) OR "hiv aids"[All Fields] | 162670 |
| #9 | **hiv infection:** "hiv infections"[MeSH Terms] OR ("hiv"[All Fields] AND "infections"[All Fields]) OR "hiv infections"[All Fields] OR ("hiv"[All Fields] AND "infection"[All Fields]) OR "hiv infection"[All Fields] | 360753 |
| #10 | **acquired immunodeficiency syndrome:** "acquired immunodeficiency syndrome"[MeSH Terms] OR ("acquired"[All Fields] AND "immunodeficiency"[All Fields] AND "syndrome"[All Fields]) OR "acquired immunodeficiency syndrome"[All Fields] | 94430 |
| #11 | **stds adolescents:** ("sexually transmitted diseases"[MeSH Terms] OR ("sexually"[All Fields] AND "transmitted"[All Fields] AND "diseases"[All Fields]) OR "sexually transmitted diseases"[All Fields] OR "stds"[All Fields]) AND ("adolescences"[All Fields] OR "adolescency"[All Fields] OR "adolescent"[MeSH Terms] OR "adolescent"[All Fields] OR "adolescence"[All Fields] OR "adolescents"[All Fields] OR "adolescent s"[All Fields]) | 60334 |
| #12 | **adolescent:** "adolescences"[All Fields] OR "adolescency"[All Fields] OR "adolescent"[MeSH Terms] OR "adolescent"[All Fields] OR "adolescence"[All Fields] OR "adolescents"[All Fields] OR "adolescent s"[All Fields] | 2314494 |
| #13 | **indian adolescent:** ("indian"[All Fields] OR "indian s"[All Fields] OR "indians"[All Fields]) AND ("adolescences"[All Fields] OR "adolescency"[All Fields] OR "adolescent"[MeSH Terms] OR "adolescent"[All Fields] OR "adolescence"[All Fields] OR "adolescents"[All Fields] OR "adolescent s"[All Fields]) | 32366 |
| #14 | **young adults:** "young adult"[MeSH Terms] OR ("young"[All Fields] AND "adult"[All Fields]) OR "young adult"[All Fields] OR ("young"[All Fields] AND "adults"[All Fields]) OR "young adults"[All Fields] | 1224987 |
| #15 | **adolescent young adults:** ("adolescences"[All Fields] OR "adolescency"[All Fields] OR "adolescent"[MeSH Terms] OR "adolescent"[All Fields] OR "adolescence"[All Fields] OR "adolescents"[All Fields] OR "adolescent s"[All Fields]) AND ("young adult"[MeSH Terms] OR ("young"[All Fields] AND "adult"[All Fields]) OR "young adult"[All Fields] OR ("young"[All Fields] AND "adults"[All Fields]) OR "young adults"[All Fields]) | 608298 |
| #16 | **youth:** "adolescent"[MeSH Terms] OR "adolescent"[All Fields] OR "youth"[All Fields] OR "youths"[All Fields] OR "youth s"[All Fields] | 2307483 |
| #17 | **teenager:** "adolescent"[MeSH Terms] OR "adolescent"[All Fields] OR "teenage"[All Fields] OR "teenager"[All Fields] OR "teenagers"[All Fields] OR "teenaged"[All Fields] OR "teenager s"[All Fields] OR "teenages"[All Fields] | 2271620 |
| #18 | **indian:** "indian"[All Fields] OR "indian s"[All Fields] OR "indians"[All Fields] | 353691 |
| #19 | **india:** "india"[MeSH Terms] OR "india"[All Fields] OR "india s"[All Fields] OR "indias"[All Fields] | 726761 |
| #20 | **Maharashtra:** "Maharashtra"[All Fields] | 30,571 |
| #21 | **New Delhi:** delhi, new[Investigator] | 2 |
| #22 | **Gujarat:** "gujarat"[All Fields] | 15,373 |
| (#1 OR #5 OR #6 OR #7) AND #2 AND #3 AND #4 AND #7 AND (#8 OR #9 OR #10 OR #11) AND (#12 OR #13 OR #14 OR #15 AND #16 OR #17) AND (#18 OR #19 OR #20 OR #21 OR #22) | **(knowledge OR knowledge attitude OR knowledge attitude practice OR health knowledge attitudes practice) AND health beliefs AND health attitudes AND health practices AND health knowledge attitudes practice AND (hiv aids OR hiv infection OR acquired immunodeficiency syndrome OR stds adolescents) AND (adolescent OR indian adolescent OR young adults OR adolescent young adults OR youth OR teenager) AND (indian OR india OR Gujarat OR Maharashtra OR New Delhi)):**  ("knowledge"[MeSH Terms] OR "knowledge"[All Fields] OR "knowledge s"[All Fields] OR "knowledgeability"[All Fields] OR "knowledgeable"[All Fields] OR "knowledgeably"[All Fields] OR "knowledges"[All Fields] OR (("knowledge"[MeSH Terms] OR "knowledge"[All Fields] OR "knowledge s"[All Fields] OR "knowledgeability"[All Fields] OR "knowledgeable"[All Fields] OR "knowledgeably"[All Fields] OR "knowledges"[All Fields]) AND ("attitude"[MeSH Terms] OR "attitude"[All Fields] OR "attitudes"[All Fields] OR "attitude s"[All Fields])) OR (("knowledge"[MeSH Terms] OR "knowledge"[All Fields] OR "knowledge s"[All Fields] OR "knowledgeability"[All Fields] OR "knowledgeable"[All Fields] OR "knowledgeably"[All Fields] OR "knowledges"[All Fields]) AND ("attitude"[MeSH Terms] OR "attitude"[All Fields] OR "attitudes"[All Fields] OR "attitude s"[All Fields]) AND ("practicability"[All Fields] OR "practicable"[All Fields] OR "practical"[All Fields] OR "practicalities"[All Fields] OR "practicality"[All Fields] OR "practically"[All Fields] OR "practicals"[All Fields] OR "practice"[All Fields] OR "practice s"[All Fields] OR "practiced"[All Fields] OR "practices"[All Fields] OR "practicing"[All Fields])) OR ("health knowledge, attitudes, practice"[MeSH Terms] OR ("health"[All Fields] AND "knowledge"[All Fields] AND "attitudes"[All Fields] AND "practice"[All Fields]) OR "practice attitudes health knowledge"[All Fields] OR ("health"[All Fields] AND "knowledge"[All Fields] AND "attitudes"[All Fields] AND "practice"[All Fields]) OR "health knowledge attitudes practice"[All Fields])) AND (("health"[MeSH Terms] OR "health"[All Fields] OR "health s"[All Fields] OR "healthful"[All Fields] OR "healthfulness"[All Fields] OR "healths"[All Fields]) AND ("belief s"[All Fields] OR "culture"[MeSH Terms] OR "culture"[All Fields] OR "belief"[All Fields] OR "beliefs"[All Fields])) AND ("attitude to health"[MeSH Terms] OR ("attitude"[All Fields] AND "health"[All Fields]) OR "attitude to health"[All Fields] OR ("health"[All Fields] AND "attitudes"[All Fields]) OR "health attitudes"[All Fields]) AND (("health"[MeSH Terms] OR "health"[All Fields] OR "health s"[All Fields] OR "healthful"[All Fields] OR "healthfulness"[All Fields] OR "healths"[All Fields]) AND ("practicability"[All Fields] OR "practicable"[All Fields] OR "practical"[All Fields] OR "practicalities"[All Fields] OR "practicality"[All Fields] OR "practically"[All Fields] OR "practicals"[All Fields] OR "practice"[All Fields] OR "practice s"[All Fields] OR "practiced"[All Fields] OR "practices"[All Fields] OR "practicing"[All Fields])) AND ("health knowledge, attitudes, practice"[MeSH Terms] OR ("health"[All Fields] AND "knowledge"[All Fields] AND "attitudes"[All Fields] AND "practice"[All Fields]) OR "practice attitudes health knowledge"[All Fields] OR ("health"[All Fields] AND "knowledge"[All Fields] AND "attitudes"[All Fields] AND "practice"[All Fields]) OR "health knowledge attitudes practice"[All Fields]) AND ("curr opin hiv aids"[Journal] OR ("hiv"[All Fields] AND "aids"[All Fields]) OR "hiv aids"[All Fields] OR ("hiv infections"[MeSH Terms] OR ("hiv"[All Fields] AND "infections"[All Fields]) OR "hiv infections"[All Fields] OR ("hiv"[All Fields] AND "infection"[All Fields]) OR "hiv infection"[All Fields]) OR ("acquired immunodeficiency syndrome"[MeSH Terms] OR ("acquired"[All Fields] AND "immunodeficiency"[All Fields] AND "syndrome"[All Fields]) OR "acquired immunodeficiency syndrome"[All Fields]) OR (("sexually transmitted diseases"[MeSH Terms] OR ("sexually"[All Fields] AND "transmitted"[All Fields] AND "diseases"[All Fields]) OR "sexually transmitted diseases"[All Fields] OR "stds"[All Fields]) AND ("adolescences"[All Fields] OR "adolescency"[All Fields] OR "adolescent"[MeSH Terms] OR "adolescent"[All Fields] OR "adolescence"[All Fields] OR "adolescents"[All Fields] OR "adolescent s"[All Fields]))) AND ("adolescences"[All Fields] OR "adolescency"[All Fields] OR "adolescent"[MeSH Terms] OR "adolescent"[All Fields] OR "adolescence"[All Fields] OR "adolescents"[All Fields] OR "adolescent s"[All Fields] OR (("indian"[All Fields] OR "indian s"[All Fields] OR "indians"[All Fields]) AND ("adolescences"[All Fields] OR "adolescency"[All Fields] OR "adolescent"[MeSH Terms] OR "adolescent"[All Fields] OR "adolescence"[All Fields] OR "adolescents"[All Fields] OR "adolescent s"[All Fields])) OR ("young adult"[MeSH Terms] OR ("young"[All Fields] AND "adult"[All Fields]) OR "young adult"[All Fields] OR ("young"[All Fields] AND "adults"[All Fields]) OR "young adults"[All Fields]) OR (("adolescences"[All Fields] OR "adolescency"[All Fields] OR "adolescent"[MeSH Terms] OR "adolescent"[All Fields] OR "adolescence"[All Fields] OR "adolescents"[All Fields] OR "adolescent s"[All Fields]) AND ("young adult"[MeSH Terms] OR ("young"[All Fields] AND "adult"[All Fields]) OR "young adult"[All Fields] OR ("young"[All Fields] AND "adults"[All Fields]) OR "young adults"[All Fields])) OR ("adolescent"[MeSH Terms] OR "adolescent"[All Fields] OR "youth"[All Fields] OR "youths"[All Fields] OR "youth s"[All Fields]) OR ("adolescent"[MeSH Terms] OR "adolescent"[All Fields] OR "teenage"[All Fields] OR "teenager"[All Fields] OR "teenagers"[All Fields] OR "teenaged"[All Fields] OR "teenager s"[All Fields] OR "teenages"[All Fields])) AND ("indian"[All Fields] OR "indian s"[All Fields] OR "indians"[All Fields] OR ("india"[MeSH Terms] OR "india"[All Fields] OR "india s"[All Fields] OR "indias"[All Fields]) OR "Gujarat"[All Fields] OR "Maharashtra"[All Fields] OR delhi, new[Investigator]) | 51 |

**2: Draft of search strategy to be used in EMBASE electronic database**

| **Components** | **Search items** | **Results** |
| --- | --- | --- |
| #1 | **knowledge:** ‘knowledge’/exp OR knowledge | 1,234,151 |
| #2 | **health belief:** ‘health belief’/exp OR ‘health belief’ | 17,918 |
| #3 | **health attitude:** ‘health attitude’/exp OR ‘health attitude’ OR ((‘health’/exp OR health) AND (‘attitude’/exp OR attitude)) | 609,988 |
| #4 | **health practices:** ‘health practices’ OR ((‘health’/exp OR health) AND practices) | 222,285 |
| #5 | **knowledge attitude practice:** ‘knowledge attitude practice’ OR ((‘knowledge’/exp OR knowledge) AND (‘attitude’/exp OR attitude) AND (‘practice’exp OR practice)) | 58,370 |
| #6 | **health knowledge attitude practices:** 'health knowledge attitude practice' OR (('health'/exp OR health) AND ('knowledge'/exp OR knowledge) AND ('attitude'/exp OR attitude) AND ('practice'/exp OR practice)) | 52,532 |
| #7 | **hiv aids:** ‘hiv aids’/exp OR ‘hiv aids’ OR ((‘hiv’/exp OR hiv) AND (‘aids’/exp OR aids)) | 208,617 |
| #8 | **hiv infection:** ‘hiv infection’/exp OR ‘hiv infection’ OR ((‘hiv’/exp OR hiv) AND (‘infection’/exp OR infection)) | 516,584 |
| #9 | **acquired immune deficiency syndrome:** ‘acquired immune deficiency syndrome’/exp OR ‘acquired immune deficiency syndrome’ | 155,070 |
| #10 | **sexually transmitted disease:** ‘sexually transmitted disease’/exp OR ‘sexually transmitted disease’ | 119,925 |
| #11 | **adolescent:** ‘adolescent’/exp OR adolescent | 1,988,853 |
| #12 | **indian adolescent:** ‘indian adolescent’ OR ((‘indian’/exp OR indian) AND (‘adolescent’/exp OR adolescent)) | 35,617 |
| #13 | **young adult:** ‘young adult’/exp OR ‘young adult’ | 514,837 |
| #14 | **indian:** ‘indian’/exp OR indian | 486,111 |
| #15 | **india:** ‘india’/exp OR india | 1,313,565 |
| #16 | **new delhi:** ‘new delhi’ OR (new AND delhi) | 168,838 |
| #17 | **maharashtra:** ‘maharashtra’/exp OR maharashtra | 46,522 |
| #18 | **gujarat:** ‘gujarat’/exp OR gujarat | 25,135 |
| #19 | **(#5 OR #6) AND (#7 OR #8 OR #9) AND (#11 OR #12 OR #13) AND (#15 OR #14 OR #16 OR #17 OR #18)** | 47 |
